# Pneumococcal colonization and coinfecting respiratory viruses in children under 5 years in Addis Ababa, Ethiopia: a prospective case–control study

**DOI:** 10.1038/s41598-024-54256-w

**Published:** 2024-02-20

**Authors:** Fiseha Wadilo Wada, Adey Feleke Desta, Meseret Gebre, Wude Mihret, Tamrayehu Seyoum, Kalkidan Melaku, Ashenafi Alemu, Rawleigh Howe, Andargachew Mulu, Adane Mihret

**Affiliations:** 1 Armauer Hansen Research Institute, Ministry of Health, Addis Ababa, Ethiopia; 2 Department of Biomedical Sciences, College of Natural and Computational Sciences, Addis Ababa University, Addis Ababa, Ethiopia; 3 Department of Medical Laboratory Sciences, College of Health Sciences and Medicine, Wolaita Sodo University, Wolaita Soddo, Ethiopia; 4 Department of Microbiology, Immunology, and Parasitology, School of Health Sciences, Addis Ababa University, Addis Ababa, Ethiopia

**Keywords:** *Streptococcus pneumoniae*, Lower respiratory tract infections, Respiratory viruses, Children, Ethiopia, Microbiology, Molecular biology

## Abstract

A comprehensive understanding of the dynamics of *Streptococcus pneumoniae* colonization in conjunction with respiratory virus infections is essential for enhancing our knowledge of the pathogenesis and advancing the development of effective preventive strategies. Therefore, a case–control study was carried out in Addis Ababa, Ethiopia to investigate the colonization rate of *S. pneumoniae* and its coinfection dynamics with respiratory viruses among children under the age of 5 years. Samples from the nasopharyngeal and/or oropharyngeal, along with socio-demographic and clinical information, were collected from 420 children under 5 years old (210 cases with lower respiratory tract infections and 210 controls with conditions other than respiratory infections.). A one-step Multiplex real-time PCR using the Allplex Respiratory Panel Assays 1–4 was performed to identify respiratory viruses and bacteria. Data analysis was conducted using STATA software version 17. The overall colonization rate of *S. pneumoniae* in children aged less than 5 years was 51.2% (215/420). The colonization rates in cases and controls were 54.8% (115/210) and 47.6% (100/210), respectively (*p* = 0.14). Colonization rates were observed to commence at an early age in children, with a colonization rate of 48.9% and 52.7% among infants younger than 6 months controls and cases, respectively. The prevalence of AdV (OR, 3.11; 95% CI [1.31–8.19]), RSV B (OR, 2.53; 95% CI [1.01–6.78]) and HRV (OR, 1.7; 95% CI [1.04–2.78]) tends to be higher in children who tested positive for *S. pneumoniae* compared to those who tested negative for *S. pneumoniae*. Further longitudinal research is needed to understand and determine interaction mechanisms between pneumococci and viral pathogens and the clinical implications of this coinfection dynamics.

## Introduction

Acute Lower Respiratory Infections (ALRIs) remain one of the leading causes of morbidity and mortality among children under 5 years old globally, with sub-Saharan Africa bearing a disproportionate burden^1^. *Streptococcus pneumoniae* is recognized as a primary pathogen responsible for ALRIs accounting for a higher number of deaths compared to all other etiologies combined, and contributes to a staggering total of 1,189,937 deaths in 2016 in people of all ages, worldwide^2^.

Colonization of the nasopharynx by *S. pneumoniae* is considered to be a prerequisite to pneumococcal disease development and transmission within communities^3,4^. The introduction of pneumococcal conjugate vaccines (PCV) has had a notable influence on the colonization dynamics of *S. pneumoniae*; numerous research studies indicate that the introduction of PCV can result in shifts in the prevalence of circulating serotypes, as non-vaccine serotypes may emerge or become more prevalent following a reduction in targeted serotypes due to vaccination-induced effects^5–8^.

In recent years, there has been growing interest in understanding the coinfection dynamics between *S. pneumoniae* colonization and respiratory viruses^9–12^. Coinfection by pneumococci and respiratory viruses often leads to increased disease severity^10^. As an example, it is widely believed that a significant number of fatalities during the 1918–1919 influenza pandemic were primarily caused by secondary bacterial pneumonia, with *S. pneumoniae* being the most prevalent bacteria associated with these cases^13^. It is hypothesized that interactions between pneumococci and respiratory viruses could potentially amplify pathogenicity or modify immune responses, thereby contributing to the development of more severe symptoms or complications^12^.

In a previous report, we highlighted the notable impact of RSV, Influenza A, and PIV 1 viruses on acute lower respiratory infections (ALRIs) among children under 5 years in Addis Ababa, Ethiopia^14^. Understanding the dynamics of *S. pneumoniae* colonization alongside respiratory virus infections is crucial for improving our knowledge about pathogenesis and developing effective preventive strategies such as vaccines or antiviral therapies. The objective of this study was to investigate *S. pneumoniae* colonization rate and the coinfection dynamics with respiratory viruses in children under 5 years old.

## Materials and methods

### Study area and period

A prospective case–control study was conducted in Addis Ababa, Ethiopia, from September 2019 to May 2022, involving two major governmental hospitals: St. Paul Hospital Millennium Medical College and ALERT Comprehensive Specialized Hospital. However, data collection was temporarily halted from February 2020 to July 2020 due to the COVID-19 pandemic.

### Study population

The case population consisted of children under 5 years old with LRTIs, defined as an acute respiratory illness with a history of fever or measured fever of ≥ 38 °C and cough within the past 10 days that required hospitalization. Controls were also children under five who were admitted to the same hospital for conditions other than respiratory infections such as injury, skin infections, gastrointestinal infections, or genitourinary infections. Participants who were above 60 months of age were excluded from both the cases and controls. Additionally, children with ARTIs that had an onset beyond 10 days were also excluded from the cases. Throughout the study period, both cases and controls were enrolled based on age group and month of sample collection using marginal frequency matching techniques.

### Data and naso/oropharyngeal swab collection

Experienced pediatric nurses identified eligible cases and controls. They obtained informed consent from parents/guardians, collected sociodemographic and clinical information, and gathered samples using naso/oropharyngeal swabs.

Nasopharyngeal and oropharyngeal swabs were obtained from all the children participating in the study. Nasopharyngeal specimens were obtained by inserting flocked swabs (Copan) into the posterior nasopharynx and rotating 180° for 2–3 s. Subsequently, oropharyngeal specimens were collected using MWE Swabs, targeting both tonsillar pillars and the posterior oropharynx for several seconds^15^. Following collection, the swabs from both sites were placed together in a 3-mL vial containing universal transport media (SIGMA VCM). Then the samples were transported to the Armauer Hansen Research Institute (AHRI) and stored at − 80 °C until further testing.

### Laboratory procedures

The laboratory procedures were conducted as described in our previous report^14^. In summary, nucleic acid extraction from naso/oropharyngeal samples was performed manually using the Ribospin_vRD Viral RNA/DNA Extraction kit (GeneAll, South Korea) following the manufacturer's protocol. Subsequently, respiratory virus detection was carried out using Allplex Respiratory Panel 1–3 Assays for viral detection and Panel 4 for bacterial detection (Seegene, South Korea) (Table 1). Amplification was done using a CFX96 thermocycler (BioRad, Hercules CA, USA), and PCR setup and result analysis were managed with the CFX real-time PCR detection system (CFX Manager Software-IVD v1.6). A Ct value of ≤ 42 (as determined by the manufacturer) was used to define positivity for each virus and *S. pneumoniae*.

**Table 1:** Allplex respiratory panel 1–4 assays analytes.

Allplex respiratory Panel 1	Allplex respiratory Panel 2	Allplex respiratory Panel 3	Allplex respiratory Panel 4
Influenza A virus (Flu A)	Adenovirus (AdV)	Bocavirus 1/2/3/4 (HBoV)	*Bordetella parapertussis* (BPP)
Influenza A-H1 (Flu A-H1)	Enterovirus (HEV)	Coronavirus 229E (229E)	*Bordetella pertussis* (BP)
Influenza A-H1pdm09 (Flu A-H1pdm09)	Metapneumovirus (MPV)	Coronavirus NL63 (NL63)	*Chlamydophila pneumoniae* (CP)
Influenza A-H3 (Flu A-H3)	Parainfluenza virus 1 (PIV 1)	Coronavirus OC43 (OC43)	*Haemophilus influenzae* (HI)
Influenza B virus (Flu B)	Parainfluenza virus 2 (PIV 2)	Human rhinovirus (HRV)	*Legionella pneumophila* (LP)
Respiratory syncytial virus A (RSV A)	Parainfluenza virus 3 (PIV 3)	Internal Control (IC)	*Mycoplasma pneumoniae* (MP)
Respiratory syncytial virus B (RSV B)	Parainfluenza virus 4 (PIV 4)		*Streptococcus pneumoniae* (SP)
Internal control (IC)	Internal control (IC)		Internal control (IC)

### Data analysis

The data analysis was conducted using Stata software version 17. To examine the characteristics of the *S. pneumoniae* positive and negative population, as well as to assess the coinfection dynamics with respiratory viruses, odds ratios (OR) were calculated. Additionally, the mean Cycle threshold (CT) values for each detected virus were determined and the Two-sample Wilcoxon rank-sum (Mann–Whitney) test was used to investigate any associations between viral load and the presence of *S. pneumoniae* in the upper respiratory tract (URT).

### Ethical review and regulation

The ethical review committees of AHRI and Addis Ababa University granted approval for this study. Prior to enrollment, written informed consent was obtained from all parents or guardians of the participating children, which was documented through their signatures.

All methods and processing of personal data strictly adhered to the Armauer Hansen Research Institute establishment regulation no. 530/2023 and Federal Civil Servants Proclamation No. 1064/2017 issued by the council of ministers of the federal government of Ethiopia.

### Ethics statement

Approval for this study was granted by the ethical review committees of AHRI and Addis Ababa University. Before enrolling participants, written informed consent was obtained from the parents or guardians of the children involved. This consent was documented through their signatures.

## Results

### Prevalence of pneumococcal colonization rate

The overall colonization rate of *S. pneumoniae* in children aged less than 5 years was 51.2% (215/420). The colonization rates in cases and controls were 54.8% (115/210) and 47.6% (100/210), respectively (*p* = 0.14). Very few samples tested positive for other respiratory bacteria in both the case and control groups (Table 2). Table 3 provides the characteristics of both the population colonized and non-colonized by *S. pneumoniae*. Generally, there is no statistically significant difference between the two groups.

**Table 2:** The prevalence of bacteria detected in the naso/oropharyngeal of under five children with and without ALRIs.

Bacteria	Case (n = 210) (%)	Control (n = 210) (%)
*Streptococcus pneumoniae*	115 (54.8)	47.6 (100)
*Legionella pneumophila*	1 (0.5)	0 (0)
*Bordetella parapertussis*	1 (0.5)	2 (1.0)
*Mycoplasma pneumoniae*	1 (0.5)	1 (0.5)
*Bordetella pertussis*	1 (0.5)	0 (0)
*Chlamydophila pneumoniae*	0 (0)	2 (1.0)
*Haemophilus influenzae*	9 (4.3)	7 (3.3)
Negative for all tested bacteria	94 (44.8)	108 (51.2)

**Table 3:** Characteristics of *S. pneumoniae* positive and negative population.

Characteristic	*S. pneumoniae* Positive (n = 215)	*S. pneumoniae* Negative (n = 205)	*P* value
Sex (Male)	114 (52.9%)	94 (45.7%)	0.052
Mean age in month	16.1 (SD = 14.4)	18 (SD = 16.2)	0.268
Mean admission weight (KG)	8.8 (SD = 3.2)	8.9 (SD = 3.7)	0.626
Mean admission height (CM)	68.0 (SD = 17.5)	71.9 (SD = 18.5)	0.075
Mean Mid upper arm circumference (CM)	13.7 (SD = 5.2)	12.7 (SD = 1.4)	0.011
Malnutrition status			
Normal	190 (88.6%)	183 (89.4%)	
Moderate acute malnutrition (MAM)	12 (5.7%)	10 (5%)	0.772
Severe acute malnutrition (SAM)	12 (5.7%)	11 (5.6%)	0.945
Breastfeeding practices	186 (86.5%)	187 (91.0%)	0.192
HIV/AIDS positive	3 (1.2%)	0	0.086
Immunization			
Immunized all scheduled program	131 (60.9%)	138 (67.2%)	
Miss some immunization	10 (4.5%)	10 (5%)	0.979
Not immunized at all	14 (6.7%)	9 (4.4%)	0.283
Immunization on progress (for < 9 months children)	60 (27.9%)	48 (23.3%)	0.260
Mean child’s mother age	29.2 (SD = 4.7)	28.5 (SD = 4.0)	0.121
Maternal education			
Illiterate	44 (20.6%)	46 (22.6%)	
Non-formal education	3 (1.2%)	1 (0.6%)	0.534
Primary School	101 (47.1%)	91 (44.6%)	0.605
Secondary School	42 (19.4%)	37 (17.9%)	0.606
Higher Education	25 (11.8%)	29 (14.3%)	0.794
Smokers in household			
Yes	10 (4.6%)	2 (1.1%)	0.092

SD, Standard deviation.

**Figure 1:**
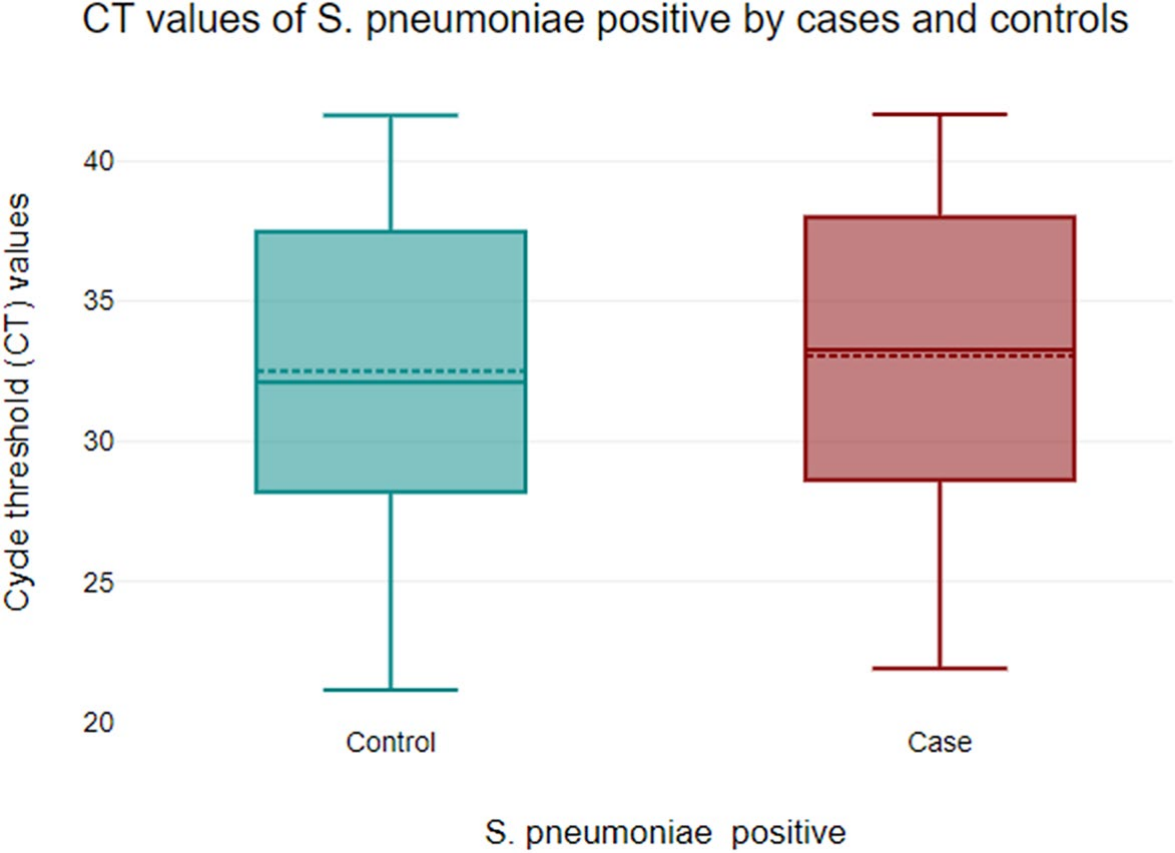
A box plot of mean/median cycle threshold (CT) values of *S. pneumoniae* colonization in the cases and control group. NB: In the boxes the broken line (-------) represents mean and the unbroken line (_____) represents the median.

The mean Cycle threshold (CT) values were compared between individuals who tested positive for *S. pneumoniae* colonization in the cases and control groups, serving as an indirect measure of bacterial load (Fig. 1). The Two-sample Wilcoxon rank-sum (Mann–Whitney) test did not show any statistically significant differences in the mean CT values between the two groups (*p* = 0.5). Furthermore, the analysis of the percentage of pneumococcal positive samples with CT values < 35, between 35 and 40, and > 42 did not reveal any significant statistical difference between the case and control groups (*p* = 0.84) (Table 4).

**Table 4:** Pneumococcal positive samples Cycle threshold (CT) values between the case and control group.

*S. pneumoniae* Positive	Cycle threshold (CT) values			Total
	< 35(n, %)	35–40(n, %)	> 40(n, %)	
Case	72 (62.6)	30 (26.1)	13 (11.3)	115
Control	63 (63)	28 (28)	9 (9)	100

### Colonization rates of *S. pnuemoniae* across ages

**Figure 2:**
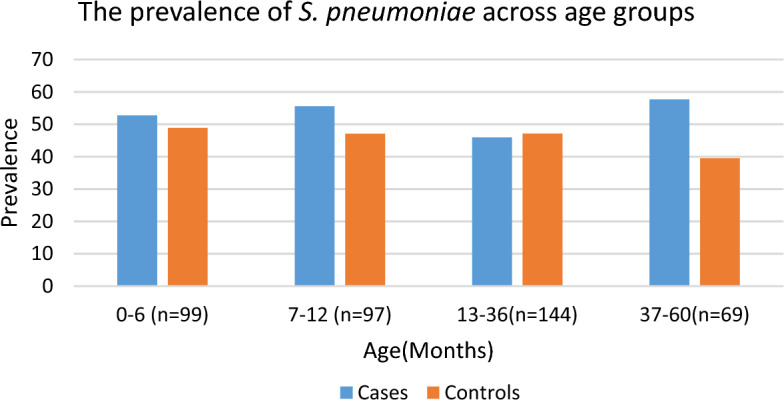
Colonization rates of *S. pnuemoniae* across ages.

The colonization rate across the different age groups of the control population ranges between 40 and 49%; whereas in the case groups, it ranges between 53 and 58% (Fig. 2). No statistically significant differences (*p* > 0.05) were observed in the colonization rates between children in the cases and control groups across different age groups. Colonization rates of *S. pneumoniae* were observed to commence at an early age in children, with a colonization rate of 22 (48.9%) and 29 (52.7%) among infants younger than 6 months controls and cases, respectively.

### Prevalence of viruses in children tested positive and negative for *S. pneumoniae*

**Figure 3:**
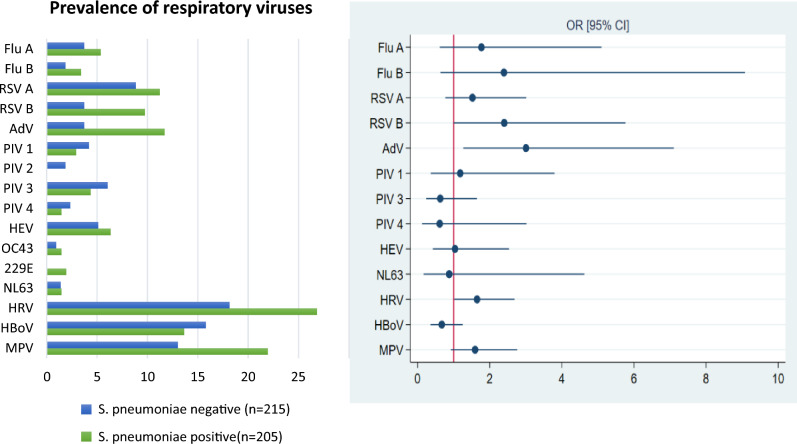
Prevalence of viruses in children who tested positive for *S. pneumoniae* compared to those who tested negative [Left] ORs with 95% CIs for detection of respiratory viruses in *S. pneumoniae* positive children compared with negatives [Right]. Flu A, Influenza A virus; Flu B, Influenza B virus; RSV A, Respiratory syncytial virus A; RSV B, Respiratory syncytial virus B; AdV, Adenovirus; PIV 1, Parainfluenza virus 1; PIV 3, Parainfluenza virus 3; PIV 4, Parainfluenza virus 4; HEV, Enterovirus; NL63, Coronavirus NL63; HRV, Human rhinovirus; HBoV, Bocavirus 1/2/3/4; MPV, Metapneumovirus.

The prevalence of AdV (OR, 3.11; 95% CI [1.31–8.19]), RSV B (OR, 2.53; 95% CI [1.01–6.78]) and HRV (OR, 1.7; 95% CI [1.04–2.78]) tends to be higher in children who tested positive for *S. pneumoniae* compared to those who tested negative for *S. pneumoniae* (Fig. 3). These results indicate a significant association between Adenovirus, RSV B and HRV infections with *S. pneumoniae* colonization in children under 5 years old in the study population.

**Figure 4:**
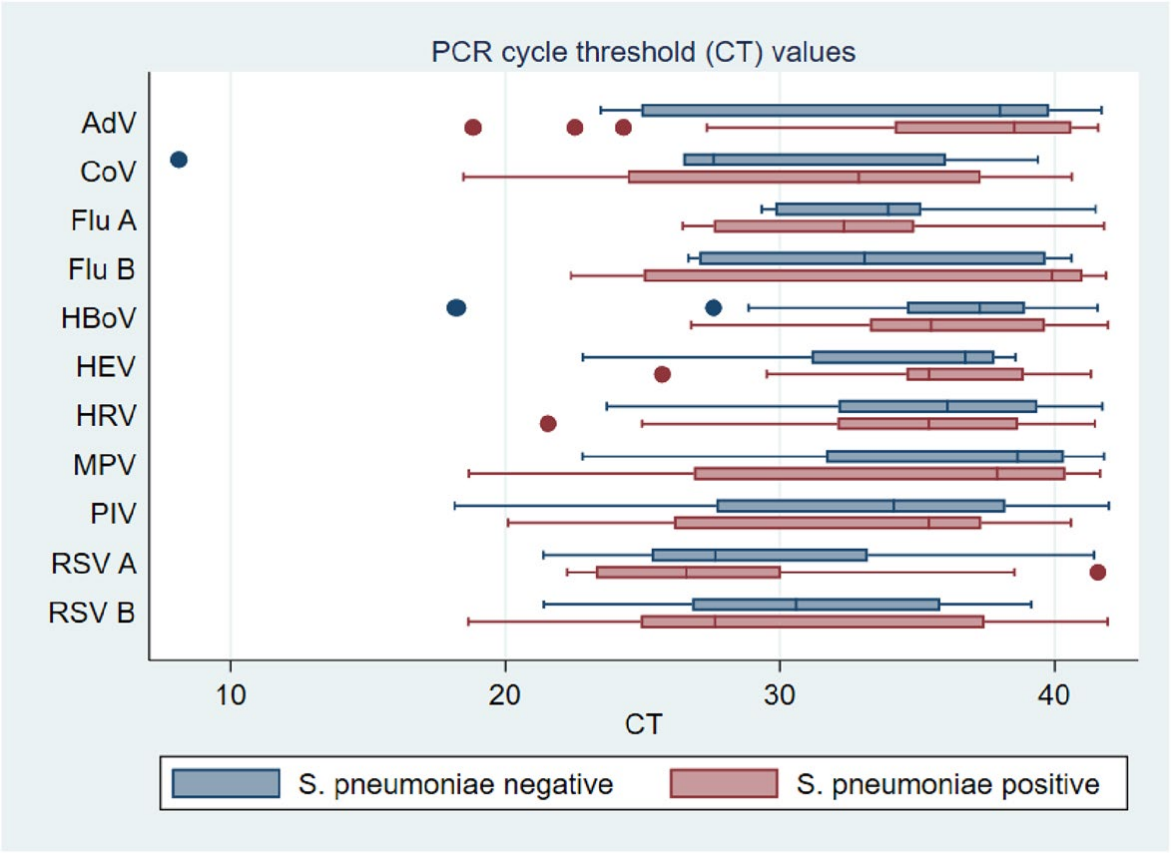
A box plot of Mean Cycle threshold (CT) values for each detected virus (**A**). Flu A, Influenza A virus; Flu B, Influenza B virus; RSV A, Respiratory syncytial virus A; RSV B, Respiratory syncytial virus B; AdV, Adenovirus; PIV 1, Parainfluenza virus 1, 2, 3, and 4; HEV, Enterovirus; CoV, Coronavirus NL63, OC43, and 229E; HRV, Human rhinovirus; HBoV, Bocavirus 1/2/3/4; MPV, Metapneumovirus NB: the line crossed the box represent the mean CT value, the dots in the figures are the outliers.

The mean Cycle threshold (CT) values for each detected virus were compared between individuals who tested positive and negative for *S. pneumoniae* colonization, serving as an indirect measure of viral load (Fig. 4). The p values calculated using the Two-sample Wilcoxon rank-sum (Mann–Whitney) test did not show any statistically significant differences in the mean CT values between the two groups (*p* = 0.50).

### Seasonality of *S. pneumoniae* and respiratory viruses

**Figure 5:**
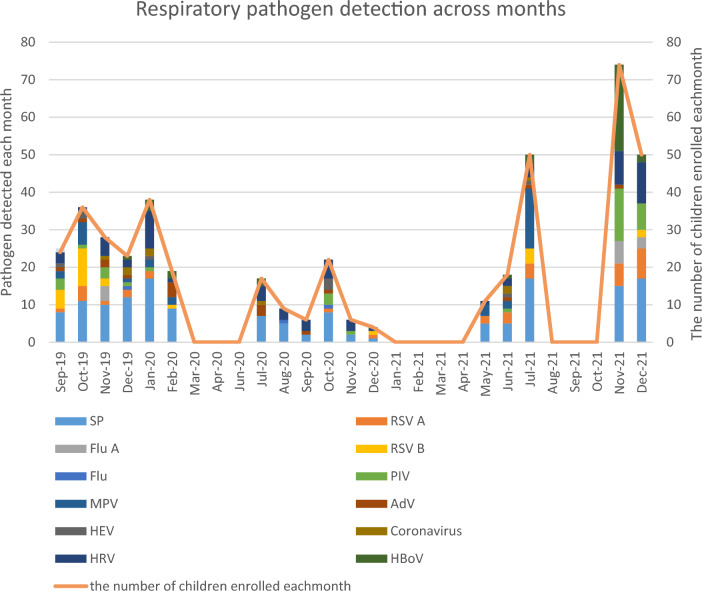
Seasonal patterns of *S. pneumoniae* and respiratory viruses in Addis Ababa, Ethiopia. SP, *S. pneumoniae*; Flu A, Influenza A virus; Flu B, Influenza B virus; RSV A, Respiratory syncytial virus A; RSV B, Respiratory syncytial virus B; AdV, Adenovirus; PIV, Parainfluenza virus 1, 2, 3, and 4; HEV, Enterovirus; CoV, Coronavirus; HRV, Human rhinovirus; HBoV, Bocavirus 1/2/3/4; MPV, Metapneumovirus.

Our finding indicates that *S. pneumoniae* and respiratory viruses occur throughout the year in Addis Ababa, Ethiopia (Fig. 5). Viral coinfection was observed frequently in the samples, with 199 (47.4%). However, in the graph, we included the detection rates of each virus, whether they were detected as a single infection or as part of a coinfection. In the first two months of sample collection (September and October 2019), there was notably a high prevalence of RSV B. The absence of data from March to June 2020 and beyond is attributed to the impact of the COVID-19 pandemic in Ethiopia. It was also observed that individual children were infected by at least two respiratory pathogens in the sampling period.

## Discussion

This study revealed that a significant portion of both cases and control children carried *S. pneumoniae* in their upper respiratory tract, 54.8% and 47.6%, respectively. In previous studies conducted in Africa, it was observed that the rate of *S. pneumoniae* detection was higher in both cases and controls; among cases, the detection rates ranged from 57.5 to 85.0%, while in control children, the detection rates varied from 48 to 88.6%^16–25^. Understanding the factors influencing the high colonization rate of *S. pneumoniae* is essential for implementing effective prevention and control strategies, such as vaccination programs and antimicrobial stewardship. Additionally, further research is necessary to explore the interactions between host factors, environmental conditions, and the bacterium itself to gain insights into the dynamics of *S. pneumoniae* colonization.

Comparing the mean CT values between the cases and controls provided information on potential variations in *S. pneumoniae* colonization levels. However, there was no significant difference detected in the mean CT values between the cases and controls of our study population. Several previous studies among children evaluated the use of pneumococcal colonization density as a marker of pneumococcal pneumonia^26–31^. Some of these studies have indicated a positive correlation between pneumococcal colonization density and severe pneumonia^26–28,31^. In contrast, other investigations did not identify any association between colonization density and the severity of pneumonia^29,30^. It is crucial to interpret these findings cautiously since additional factors such as sample size, population demographics, underlying health conditions, and antibiotic usage history may influence *S. pneumoniae* colonization dynamics and subsequent cycle threshold results. Further analysis considering these factors along with clinical outcomes will help determine whether variations observed in cycle threshold values have any meaningful associations with disease progression or prognosis within each group.

Our findings suggest that there is an association between Adenovirus and *S. pneumoniae*. Adenovirus is responsible for approximately 5–10% of ALRIs in children^32^. A study conducted in Tunisia reported that the co-detection of *S. pneumoniae* appears to exacerbate the severity of Adenovirus-associated ALRIs^33^. Furthermore, an animal model study demonstrated that adenovirus infection promotes middle ear infection caused by *S. pneumoniae* compared to non-adenovirus-infected animals^34^. Adenovirus infection may enhance the adherence of *S. pneumoniae* to mucosal cells^35^. Additional investigations are required to comprehend the underlying mechanisms of coinfection.

Our finding also indicated the presence of an association between RSV B and *S. pneumoniae* colonization in children under 5 years old in the study population, implying potential interactions and synergistic effects between these two pathogens. Numerous studies have documented interactions between *S. pneumoniae* and RSV^10,36–41^. The exact mechanisms underlying the co-infection dynamics are not fully understood; however, pneumococcus and RSV potentially have bidirectional interactions^10,41^. In vitro experiments showed enhanced adherence of *S. pneumoniae* to human epithelial cells infected with RSV^39^. Conversely, *S. pneumoniae* colonization can enhance subsequent RSV infection^41,42^. Furthermore, in a clinical trial, the administration of pneumococcal vaccination resulted in a 32% reduction in the incidence of hospitalization for pneumonia associated with RSV^43^. This co-infection dynamics highlights the need to consider both pathogens when diagnosing respiratory illnesses in young children and underscores the importance of preventive measures against both RSV B and *S. pneumoniae* infections.

One limitation of our study was the combination of nasopharyngeal and oropharyngeal swabs prior to testing. Additionally, as we used a commercial kit, the specific target gene for detecting *S. pneumoniae* was not disclosed. These factors could have potentially impacted the specificity of the test, as non-pneumococcal Streptococci present in the oral cavity may produce a false-positive signal for certain pneumococcal PCR targets^44^.

## Conclusions

The colonization rate of *S. pneumoniae* in children aged less than 5 years, with and without ALRIs, was high. Our results also indicate a significant association between Adenovirus, RSV B and HRV infections with *S. pneumoniae* colonization in the study population, implying potential interactions and synergistic effects between respiratory pathogens. Further research is needed to understand the underlying mechanisms and clinical implications of this coinfection dynamics.

## Data Availability

The datasets during and/or analyzed during the current study are available from the corresponding author upon reasonable request.
